# Neuroimaging findings in a woman with brainstem paragonimiasis: a case report and review of the literature

**DOI:** 10.1186/1752-1947-8-36

**Published:** 2014-02-05

**Authors:** Dong Yi, Dong Qiang, Cao Wenjie

**Affiliations:** 1 The Department of Neurology, Huashan Hospital, Fudan University, No. 12 Wulumuqi Zhong Rd., 200040 Shanghai, P.R. China

**Keywords:** Neuroimaging, Brainstem, Paragonimiasis

## Abstract

**Introduction:**

The most serious erratic migration of paragonimiasis is a cerebral paragonimiasis. Infection in the temporal and occipital lobes of the brain has been previously reported in cerebral paragonimiasis. Here, we report a case of chronic brainstem paragonimiasis.

**Case presentation:**

A 29-year-old Chinese woman presented to our hospital complaining of a sudden onset, three days previously, of double vision and dizziness. A computed tomography scan and diffusion-weighted images looked like an ischemic stroke. However, conglomerates of multiple ring-like enhancements on contrast magnetic resonance imaging, and the characteristic appearance of the ‘tunnel sign’, were found on further examination. Our patient was treated with praziquantel to prevent the spread of the paragonimiasis. She was followed up three months later and showed good recovery.

**Conclusions:**

Conglomerates of multiple ring-like enhancements on contrast magnetic resonance imaging and the characteristic appearance of the ‘tunnel sign’ were important for the diagnosis of chronic brainstem paragonimiasis.

## Introduction

Paragonimiasis is an infection by the genus Paragonimus, the most common of which is *Paragonimus Westermani. P. Westermani* is a common human parasite in the Far East, and is particularly prevalent in China, Japan, and Korea. Paragonimiasis results from the ingestion of raw or insufficiently cooked second intermediate hosts such as freshwater crayfish, crabs, or shrimp. The metacercariae excyst in the small intestine penetrate the wall into the abdominal cavity, which then migrate through the viscera and diaphragm to the lungs. The lung is the principal habitat in the human host. The erratic migrations of the juvenile flukes result in ectopic paragonimiasis in various organs, frequently in the peritoneal and pelvic cavities, the diaphragm, the subcutaneous tissues, and the brain [1,2]. The most serious erratic migration is a cerebral paragonimiasis, where the fluke enters the cranial cavity through the jugular or carotid foramen, and usually invades the brain. Infection of temporal and occipital lobes of the brain has been reported [2-5]. Cerebral paragonimiasis is serious and sometimes fatal, although symptoms of ectopic infections are solely dependent on the infected sites and the number of parasites. Here, we report a case of a chronic cerebral paragonimiasis in the brainstem with a sudden onset.

## Case presentation

**Figure 1:**
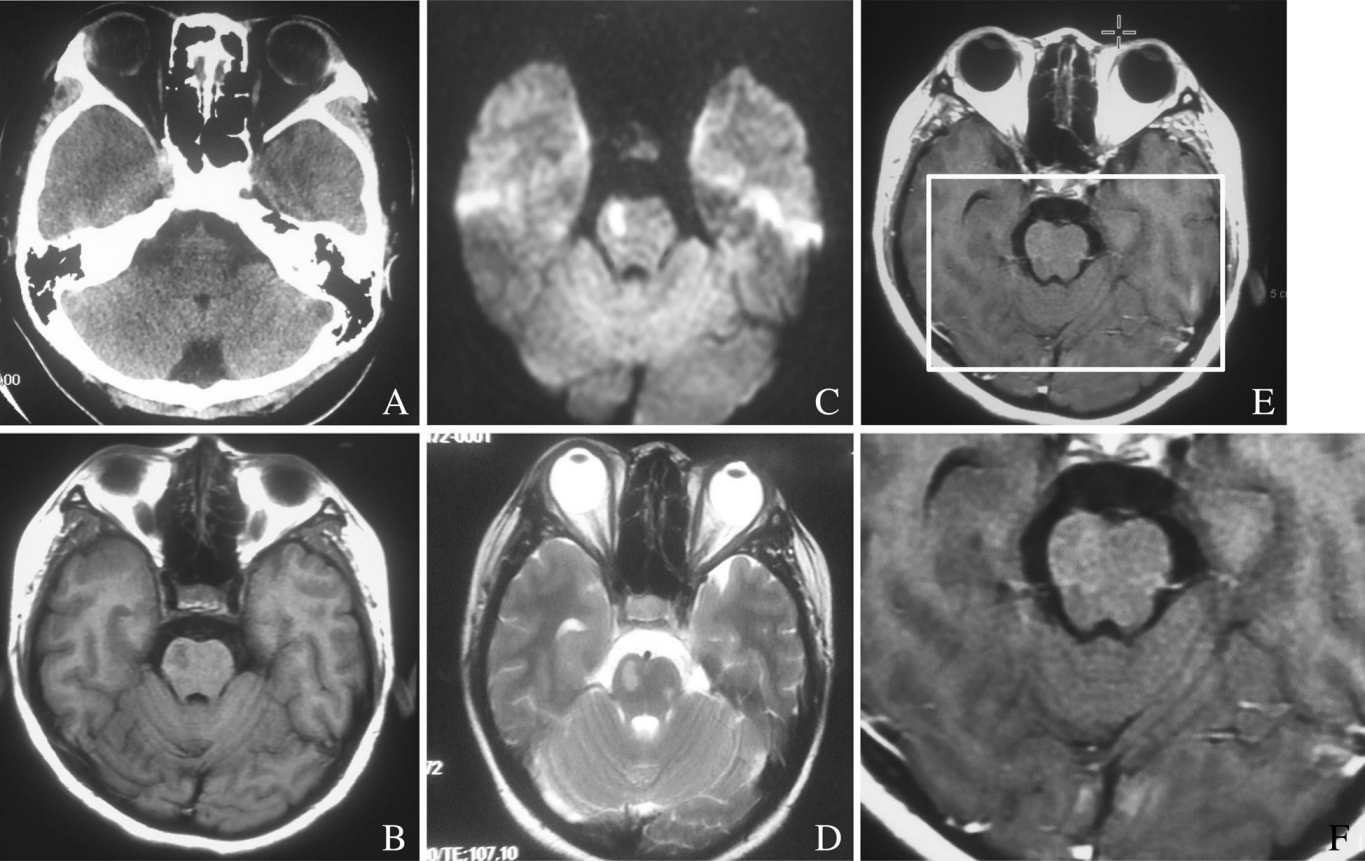
**(A) A suspect low-density lesion in the brainstem with no abnormal calcification. (B-F)** A brain magnetic resonance imaging scan revealed an egg-like lesion in the brainstem, with a slight hyperintensity in T1, hyperintensity in T2 and DWI, and conglomerates of multiple ring-like enhancements in contrast magnetic resonance imaging.

**Figure 2:**
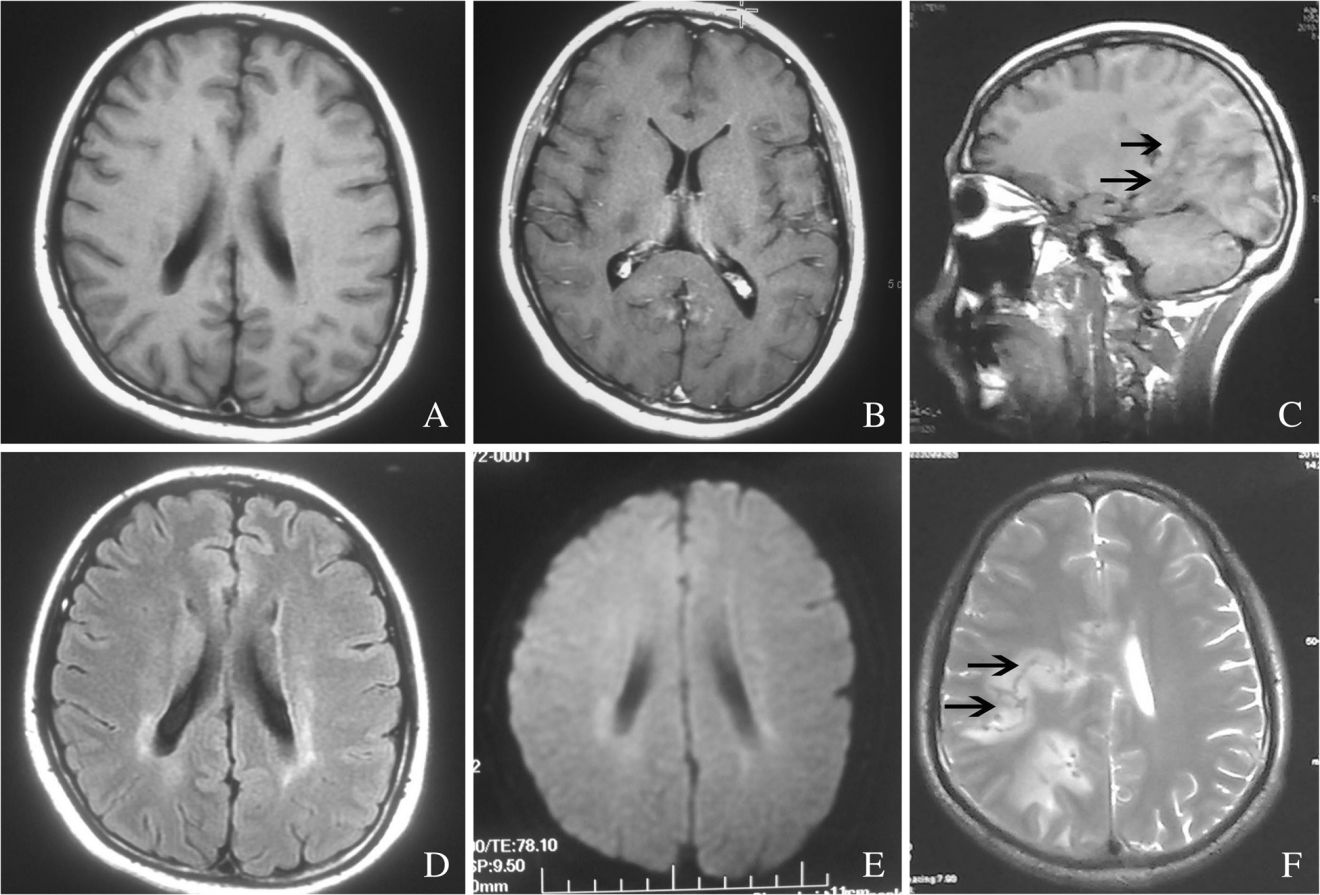
**An abnormal sign on the lateral angles on the magnetic resonance imaging scan.** The characteristic appearance was the ‘tunnel sign’, which was arrowed in T1 sagittal **(C)** and T2 axial sequences **(F)**. FLAIR imaging **(D)** indicated leukoencephalopathy on the lateral angles. However, there were no remarkable changes on T1 **(A)**, contrasted MR **(B)** and DWI imaging **(E)**.

A 29-year-old Chinese woman presented to our hospital with a sudden onset, three days previously, of double vision and a feeling of dizziness. She had vomited twice without seizures or unconsciousness. During her physical and neurological examination, a twist in her tongue and diplopia were observed. Our patient was born near an endemic area of paragonimiasis and used to eat grilled freshwater crab. The laboratory results of her blood test were: red blood cell count 4.08 × 10^12^/L, white blood cell count 4.08 × 10^9^/L (neutrophils 54.8%, lymphocytes 38.2%, monocytes 5.6%, eosinophils 1.2%, basophils 0.2%), hemoglobin 134g/L and platelet count 175 × 10^9^/L. An examination of her fecal specimens was negative and her chest radiography was normal. An emergency computed tomography (CT) scan showed a suspect low-density lesion in the brainstem with no abnormal calcification. Our patient was diagnosed as having had an ischemic stroke and treated with aspirin. However, a magnetic resonance imaging (MRI) scan of her brain revealed an egg-like lesion in her brainstem, with a slight hyperintensity on both T1 and T2, and conglomerates of multiple ring-like enhancements on contrast MRI (Figure 1). The MRI scan also revealed an abnormal signal on the lateral angles. The characteristic appearance of paragonimiasis is the ‘tunnel sign’, which shows the track of the adult worm (Figure 2). The result of a protein test of her cerebrospinal fluid was 339mg/L. The results of an enzyme-linked immunosorbent assay for *Westermani* were highly positive. Since there was no change in her blood test and no edema around the lesion, it was presumed that *P. Westermani* had formed the brain lesions a long time ago. Praziquantel (25mg/kg three times a day) was prescribed to prevent the spread of the paragonimiasis. Our patient was followed up three months later, there was no progression of the lesions, and the symptoms had disappeared.

## Discussion

The frequency of cerebral involvement has been reported in 20 to 45% of patients with erratic paragonimiasis [6]. In a recently reported case, 3.5% of patients with food-borne parasitic encephalopathy were due to cerebral paragonimiasis. In hospital-based studies, 0.8% of the positive reactions in intradermal tests for *Paragonimus* are compatible with a cerebral paragonimiasis [7]. Cerebral paragonimiasis is diagnosed by radiological findings, as well as by immunologic and parasitology methods. Nomura *et al*. reported four pathological stages of the cerebral parasite. Briefly, stage I consisted of apparently viable cysts surrounded by a thin layer of collagen type I. In stage II, a mononuclear-rich inflammatory infiltrate became evident around the parasite. At stage III, granulomas with associated inflammatory infiltrates and fibrosis were formed, and an abundant number of eosinophils had migrated to the center of the lesion, to be located between the parasite and granuloma. By stage IV the same cell types were evident, but the center of the lesion contained the disintegrated parasite and amorphous material reminiscent of necrosis [8]. This granulomatous response in swine resembled previous findings in humans, but differed in the abundance of eosinophils, the presence of fewer plasma cells and discrete deposition of collagen. These variations may be partly attributed to an immune response that is detected earlier in swine, but at a more chronic stage in humans. The CT and MRI findings for chronic cerebral paragonimiasis have been generally recorded as conglomerates of multiple ring-shaped shadows or enhancements of ‘grape cluster’ or ‘soap bubble’ forms in one hemisphere [3,4,8,9]. The present case showed typical radiology findings of chronic paragonimiasis such as multiple conglomerated round calcified nodules in the brainstem, which has not been reported. An enzyme-linked immunosorbent assay (ELISA) is recommended as a complementary tool for diagnosing cerebral paragonimiasis, and the serum and ELISA antibody levels are generally positive in 48% and 31% of chronic cases, respectively [3]. In our case, the ELISA results for the diagnosis of paragonimiasis were positive and there were many *P. Westermani* eggs in the worm capsules resected from the patients’ brain. Numerous *P. Westermani* eggs were well preserved and were also present in the tunnel sign, which showed the track of the adult worm. In addition, most of the eggs in the worm capsules retained a shell without a yolk. This indicates that the worm capsules in the brain had been established a long time ago, as adult worms die within 10 to 20 years, even without treatment [9]. In addition, the worm capsules remain as multiple nodules with a low-density cavity containing the *Paragonimus* eggs in cases of cerebral paragonimiasis that persist for more than 20 years [1,9].

## Conclusions

Our patient had a sudden onset of a chronic cerebral paragonimiasis infection. The CT scan and diffusion-weighted images resembled an ischemic stroke in the brainstem. However, conglomerates of multiple ring-like enhancements on contrast MRI, and the characteristic appearance of the ‘tunnel sign’, were important for diagnosis.

## Consent

Written informed consent was obtained from the patient for publication of this manuscript and any accompanying images. A copy of the written consent is available for review by the Editor-in-Chief of this journal.

## Abbreviations

CSF: Cerebrospinal fluid; CT: Computed tomography; DWI: Diffusion-weighted images; ELISA: Enzyme-linked immunosorbent assay; MRI: Magnetic resonance imaging; FLAIR: Fluid attenuated inversion recovery.

## Competing interests

The authors declare that they have no competing interests.

## Authors’ contributions

DY analyzed and interpreted the patient data and was a major contributor in writing the manuscript. CWJ performed the neuroimaging explanation of the cerebral lesions and helped to interpret the patient data. DQ designed and organized the study. All authors have read and approved the final manuscript.
